# Edaravone Attenuated Particulate Matter-Induced Lung Inflammation by Inhibiting ROS-NF-*κ*B Signaling Pathway

**DOI:** 10.1155/2022/6908884

**Published:** 2022-04-23

**Authors:** Yingying Zeng, Guiping Zhu, Mengchan Zhu, Juan Song, Hui Cai, Yuanlin Song, Jian Wang, Meiling Jin

**Affiliations:** Department of Pulmonary Medicine, Zhongshan Hospital, Fudan University, Shanghai 200030, China

## Abstract

**Background:**

Particulate matter (PM) exposure is related to mitochondria dysfunction and airway inflammation. Antioxidant drug edaravone (EDA) is reported to improve the occurrence and development of oxidative stress-related diseases. At present, there is no data on whether EDA can alleviate lung inflammation caused by PM.

**Methods:**

The anti-inflammatory effects of EDA were investigated in urban PM-induced human bronchial epithelial cells (HBECs) and C57/BL6J mouse models. *In vitro*, its effects on the production of intracellular reactive oxygen species (ROS), mitochondrial membrane potential (MMP), and inflammatory cytokines were assessed by DCFH-DA staining, JC-1 assay, and real-time PCR, respectively. *In vivo*, the oxidant stress in lung tissues was assessed by dihydroethidium (DHE) staining and malondialdehyde (MDA) activity, and inflammatory cytokines in bronchoalveolar lavage fluid (BALF) were assessed by ELISA, respectively. Furthermore, the potential signaling pathways were studied by siRNA transfection and western blot.

**Results:**

PM increased the expression of inflammatory cytokines and protein, including IL-6, IL-1*α*, IL-1*β*, and COX-2, while these alternations were significantly alleviated following EDA treatment in a dose-dependent manner. EDA treatment also alleviated the inflammatory responses in lung tissues of PM-exposed mice. We further showed mitochondrial dysfunction in PM-exposed HBECs and mice, which were reversed by EDA treatment. Moreover, the phosphorylation of NF-*κ*B p65 in PM-exposed HBECs and mice was weakened by EDA. Transfection with NF-*κ*B p65 siRNA further inhibited PM-induced inflammation in HBECs.

**Conclusion:**

We demonstrated that EDA treatment had a protective role in PM-induced lung inflammation through maintaining mitochondrial balance and regulating the ROS-NF-*κ*B p65 signaling pathway. This provided a new therapeutic method for PM-induced lung inflammation in the future.

## 1. Introduction

Ambient particulate matter (PM) exposure is a severe public health problem worldwide, and it is the fourth leading risk factor for global death in 2019 [1]. It was reported that 92% of the world's population were exposed to PM_2.5_ levels far exceeding the average PM_2.5_ transition level of the World Health Organization's Air Quality Guidelines. Furthermore, PM was reported to induce adverse effects on human health after short-term and long-term exposure. Short-term exposure generally causes acute inflammatory responses in airways and peripheral blood [2], while long-term exposure is actively related to the mortality of cardiovascular diseases, respiratory diseases, and cancer [3–5]. The increasing burden of PM exposure makes it urgent to control airway diseases caused by PM exposure. Accumulating epidemiological data have proved that short-term PM exposure contributed to the occurrence and development of airway inflammation and mitochondria dysfunction. However, the specific target of interventions is still unclarified.

Mitochondria are sensitive targets of PM and the primary source of oxidative stress that leads to inflammatory responses. Dysfunction mitochondria display decreased mitochondria membrane potential (MMP) and increased ROS production. Once ROS led to a decrease in MMP and an increase in mitochondrial membrane permeability, mitochondrial proapoptotic factors would be released into the cytoplasm, triggering mitochondrial-dependent apoptosis [6]. Cumulative studies suggested that airway inflammation acted as the product of mitochondrial dysfunction [7–9]. Thus, our study attempted to explore an effective therapy to reduce PM-related mitochondrial oxidative damage and protect against lung injury.

Edaravone (3-methyl-1 phenyl-2-pyrazoline-5-one, EDA) is a powerful scavenger of hydroxyl radicals, eliminating the hydroxyl radicals that induce lipid peroxidation [10–12]. Emerging research showed that it had antioxidation and antiapoptosis effects and pointed out that EDA had a beneficial effect on diseases caused by redox imbalance in inflammatory diseases [13–16]. Zhang et al. showed that EDA protected against liver and lung injury by reducing the expression of inflammatory cytokines in animal sepsis models [17]. However, the effect of EDA on PM-induced lung injury has not been studied.

Therefore, the present study for the first time demonstrated that EDA protected PM-induced lung inflammation via regulating the ROS-NF-*κ*B signaling pathway *in vivo* and *in vitro*.

## 2. Methods

### 2.1. Reagents and Antibodies

Urban PM 1649b was purchased from NIST (Gaithersburg, MD, USA). EDA was obtained from Selleck (Houston, Texas, USA). Dihydroethidium (DHE) was obtained from Sigma-Aldrich (Saint Louis, MO, USA). 2′,7′-Dichlorofluorescein diacetate (DCFH-DA) was purchased from Beyotime (Shanghai, China). Antibodies against cyclooxygenase- (COX-) 2, phospho-NF-*κ*B p65, and NF-*κ*B p65 were purchased from Cell Signaling Technology (Danvers, MA, USA). Antibodies against GAPDH were purchased from Beyotime. Antibodies against *β*-actin were purchased from Abcam (Cambridge, MA, USA). The mRNA primers were synthesized by Shenggong Technologies (Shanghai, China). All kits for cDNA synthesis and real-time PCR (RT-PCR) detection were purchased from Yeasen Biotech (Shanghai, China). The reagents for western were purchased from Beyotime.

### 2.2. Animals and Cell Culture

Animal handling and experiments were conducted following the policies of the animal care facility of Zhongshan hospital, Fudan University. Male 8-12-week-old C57BL/6 mice were acquired from SLAC Laboratory Animals (Shanghai, China). Human bronchial epithelial cells (HBECs) were purchased from the Chinese Academy of Sciences (Shanghai, China) and cultured in RPMI 1640 (Gibco, Waltham, MA, USA) containing 10% FBS (Gibco) and 50 U/mL penicillin and streptomycin (Gibco).

For *in vivo* experiments, the acute PM-exposed mouse models were established via intratracheal instillation with 100 *μ*g PM in 30 *μ*L PBS per day for 2 consecutive days, according to our previous studies [18–20]. EDA (2, 10, or 20 mg/kg) was intraperitoneally injected 1 h prior to PM stimulation every time. PBS was administered following the same protocol to obtain the control group. For *in vitro* experiments, HBECs were treated with PM at 300 *μ*g/mL for 24 h, according to our previous studies [21, 22]. EDA (300, 400, or 500 *μ*M) were used to treat HBECs 1 h before PM stimulation. The dose of EDA for *in vitro* and *in vivo* experiments was identified according to the previously reported studies [11, 17, 23–27]. To make our results accurate, the concentration gradient of EDA treatment was selected for our study.

### 2.3. Small Interfering RNA (siRNA) Transfection

NF-*κ*B p65 siRNA was purchased from GenePharma (Shanghai, China). HBECs were transfected with NF-*κ*B p65 siRNA or control siRNA using Lipofectamine 2000 (Invitrogen, Carlsbad, CA, USA) at a concentration of 50 nM according to the manufacturer's protocol. After transfection, cells were treated with PM for 24 h. Subsequently, certain genes were analyzed by RT-PCR and western blot.

### 2.4. Real-Time PCR

RT-PCR analysis was carried out to detect the mRNA expression of COX-2, IL-6, IL-1*α*, and IL-1*β*. Total RNA extraction from HBECs was performed with the TRIzol Reagent (Invitrogen). Then, RNA was reverse transcribed and quantified by the 7500 RT-PCR system (Applied Biosystems) using the SYBR Green PCR kit (Takara, Japan). All protocols were used as we previously reported [18]. The primers used in our research are listed in Table 1.

### 2.5. Western Blot

Western blot analyses were performed as previously described [18]. Briefly, the cells and the right lung upper lobe of mice were harvested and lysed in radioimmunoprecipitation assay (RIPA) buffer containing 1% phenylmethanesulfonyl fluoride (PMSF) and 1% phosphatase inhibitors (Biotool, Houston, TX, USA). Subsequently, the homogenate was centrifuged at 12,000 g for 30 min at 4°C, and the supernatant was collected.

After cell homogenates were boiled, the proteins (30 *μ*g) were separated by SDS-PAGE and transferred to the PVDF membranes. After blocking with western blocking buffer for 1 h at room temperature, membranes were incubated with primary antibody against phospho-NF-*κ*B p65, NF-*κ*B p65, COX-2, GAPDH, or *β*-actin overnight at 4°C, respectively. Then, the membranes were washed with TBST thrice and incubated with goat anti-rabbit or goat anti-mouse horseradish peroxidase- (HRP-) conjugated secondary antibodies. The targeted proteins were detected by ECL reagents. The intensity of protein bands was quantified by Image J software (v1.8.0).

### 2.6. ROS Measurement

The DCFH-DA fluorescent dye probe was used to measure intracellular ROS production. One day after seeding, cells were pretreated with EDA (300, 400, or 500 *μ*M) for 1 h, then treated with 300 *μ*g/mL PM for another 1 h. Then, cells were washed with PBS and incubated with 10 *μ*M DCFH-DA at 37°C for 15 min in the dark. Cells were washed with PBS twice, and images were obtained using the fluorescence microscope (Olympus, Tokyo, Japan) equipped with a digital camera. For *in vivo* studies, fresh lung tissues were embedded in OCT, sectioned, and stained with DHE to detect ROS, as described previously [18]. DHE entered cells and was oxidized by superoxide to emit red fluorescence detected by the fluorescence microscope. The value of relative fluorescence intensity was measured in selected areas that contain cells and was analyzed by Image J software (v1.8.0).

### 2.7. JC-1 Assay for Mitochondrial Membrane Potential

MMP was measured using a mitochondrial-specific dual-fluorescence probe, JC-1 (Beyotime). Briefly, HBECs were seeded into 12-well plates, treated with or without EDA (300, 400, or 500 *μ*M) for 1 h, and then treated with 300 *μ*g/mL PM for another 24 h. Next, cells were washed with PBS and incubated with 10 *μ*g/mL JC-1 at 37°C in the dark for 30 min. The fluorescence was measured by a microscope. The red fluorescence represents the JC-1 monomers, while the green fluorescence represents JC-1 aggregates. The ratio of red fluorescence to green fluorescence was calculated to indicate the level of MMP.

### 2.8. Malondialdehyde (MDA) Activity

The right lung middle lobe and right lung lower lobe of mice were harvested. The tissue samples were homogenized and sonicated in 300 *μ*L RIPA buffer on ice. Then, tissue lysates were centrifuged at 12,000 g for 15 min at 4°C, and the supernatant was collected. The proteins were diluted to the appropriate concentration and subjected to the MDA assay as described in the Lipid Peroxidation MDA assay kit (Beyotime). The MDA levels were detected by Multi-Mode Microplate Readers (BioTek, Santa Barbara, CA, USA) at 532 nm.

### 2.9. H&E Staining and Immunohistochemistry (IHC)

Mice from different groups were intraperitoneally injected with avertin (Sigma-Aldrich) for euthanasia at the dose of 25 mg/kg body weight, and the left lung lobe of mice was harvested and fixed in 4% paraformaldehyde. Then, the left lung was sliced and stained with hematoxylin and eosin (H&E) staining according to the manufacturer's instructions [18]. Pathologic changes were imaged and scored as reported previously [28].

Paraffin-embedded left lung tissue sections were stained by IHC. The slices were incubated with 3% H_2_O_2_ and then were incubated with a primary antibody against COX-2 at 4°C for 12 h. The second antibody labeled with horseradish peroxidase was incubated at 37°C for 30 min, and the immunohistochemical reaction was observed according to the manufacturer's instructions with 3,3′-diaminobenzidine (DAB) as a chromogenic agent [28]. IHC staining was evaluated by ImageJ software (v1.8.0) according to the MOD value.

### 2.10. Bronchoalveolar Lavage Fluid (BALF) Collection and Inflammatory Cell Counts

Mice were intraperitoneally injected with avertin for euthanasia at the dose of 25 mg/kg body weight. After euthanasia, the chest of each mouse was opened to collect the BALF. The trachea and the whole lung were cannulated and perfused with 1 mL normal saline. A three-in and three-out pattern of main bronchial instillation was adopted, and the BALF was collected with a high recovery. The BALF was centrifuged at 800 g for 5 min at room temperature. The supernatant was collected and stored at -80°C for further analysis. Cell pellets were resuspended with 0.5 mL of PBS, and the total cell number was counted. Then, 100 *μ*L of the resuspended cells was spun to the slide, air-dried, and then fixed with 4% paraformaldehyde overnight. Cells were stained with H&E, and the number of macrophages and neutrophils in 200 cells was counted. The data were shown as 10^5^/mL BALF.

### 2.11. Enzyme-Linked Immunosorbent Assay (ELISA)

The protein expression levels of IL-6, IL-1*α*, and IL-1*β* in the BALF were measured using ELISA kits (4A Biotech, Beijing, China) according to the manufacturer's instructions [18]. Values were expressed as pg/mL and deduced from standard curves of recombinant cytokines.

### 2.12. Statistical Analyses

The GraphPad Prism 5 software (GraphPad, Inc., La Jolla, CA, USA) was used to analyze all data. The significance of differences between control and experimental values was assessed using the unpaired *t*-test or one-way analysis of variance (ANOVA). *P* < 0.05 was considered statistically significant. All values were expressed as the mean ± standard error of the mean (SEM).

## 3. Results

### 3.1. EDA Attenuated PM-Induced Inflammation in HBECs

**Figure 1:**
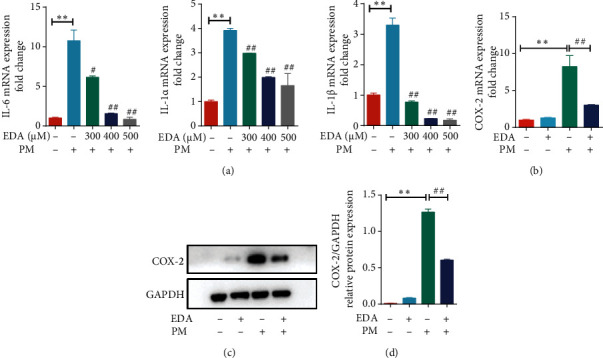
EDA attenuated PM-induced inflammation in HBECs. (a) HBECs were treated with EDA in a dose-dependent manner (300, 400, or 500 *μ*M) and then stimulated with 300 *μ*g/mL PM for 24 h. The mRNA expression of IL-6, IL-1*α*, and IL-1*β* was detected by RT-PCR. (b) The mRNA of COX-2 was detected by RT-PCR. (c) Western blot showed the expression of COX-2 in HBECs upon PM exposure after EDA treatment. The optical densities of COX-2 are shown in (d). Values are the mean ± SEM; ^∗∗^*P* < 0.01, compared with the control group; ^#^*P* < 0.05 or ^##^*P* < 0.01, compared with the PM group; *n* = 3. EDA: edaravone; PM: particulate matter; HBECs: human bronchial epithelial cells.

To investigate the anti-inflammatory effect of EDA on HBECs, the cells were treated with 300, 400, or 500 *μ*M EDA for 1 h, respectively, and then stimulated with 300 *μ*g/mL PM for an additional 24 h. The mRNA levels of inflammatory cytokines, including IL-6, IL-1*α*, and IL-1*β*, were significantly increased in HBECs with PM stimulation, and the increasing level of inflammatory cytokines was significantly decreased by EDA treatment in a dose-dependent manner in HBECs with PM stimulation (Figure 1(a)). COX-2 was induced by several inflammatory stimuli, and its expression was increased significantly by PM exposure in this study, as we previously reported [18]. EDA treatment could significantly decrease the expression of COX-2 in HBECs exposed to PM at the gene and protein levels (Figures 1(b)–1(d)). These results suggested that EDA could inhibit PM-induced inflammation in HBECs.

### 3.2. EDA Attenuated PM-Induced ROS Generation and Reversed PM-Induced MMP Dysfunction in HBECs

**Figure 2:**
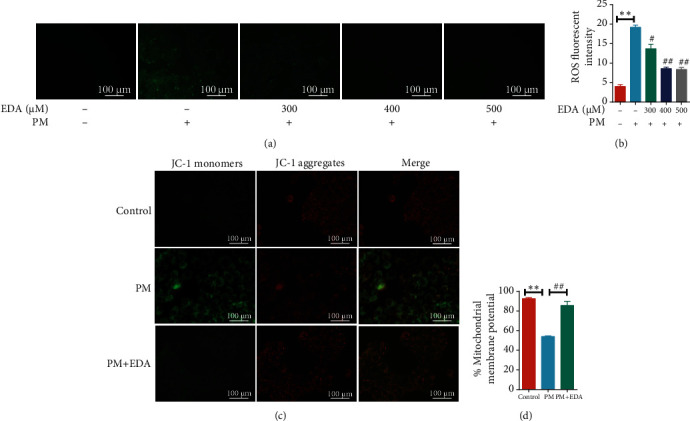
EDA inhibited PM-induced oxidative stress and mitochondrial dysfunction in HBECs. (a) HBECs were treated with EDA in a dose-dependent manner (300, 400, or 500 *μ*M) and then stimulated with 300 *μ*g/mL PM for 24 h. The ROS level was measured through the fluorescent probe, DCFH-DA, and fluorescence intensity was measured and shown in (b). (c) Mitochondrial membrane potential (*ΔΨ*m) was detected using JC-1 staining, and data are presented as the ratio of red fluorescence (JC-1 aggregates) to green fluorescence (JC-1 monomers) in (d). Values are the mean ± SEM;  ^∗∗^*P* < 0.01, compared with the control group; ^#^*P* < 0.05 or ^##^*P* < 0.01, compared with the PM group; *n* = 3. EDA: edaravone; PM: particulate matter; HBECs: human bronchial epithelial cells.

To further investigate the antioxidant role of EDA in PM-induced inflammatory responses, the ROS level of HBECs was detected in PM-treated HBECs. The ROS level in HBECs was significantly increased after PM exposure, and EDA treatment markedly reduced PM-induced oxidant stress in HBECs (Figures 2(a) and 2(b)). To address the effect of EDA on mitochondria protection, we assayed MMP using a fluorescent mitochondria dye, JC-1. Red fluorescence defines normal MMP, whereas green fluorescence defines the loss of MMP. Our results showed that MMP was significantly degraded by PM, and EDA treatment significantly restored the MMP dysfunction (Figures 2(c) and 2(d)). These results suggested that EDA attenuated oxidative stress and treated mitochondrial imbalance in HBECs exposed to PM.

### 3.3. EDA Inhibited PM-Induced Inflammation *In Vivo*

**Figure 3:**
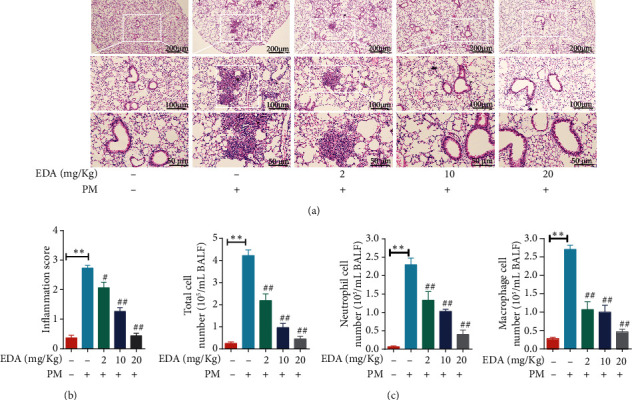
EDA alleviated the PM-induced inflammatory responses *in vivo*. Mice were intratracheally instilled with PM at 100 *μ*g/day/mouse for 2 consecutive days. EDA (2, 10, or 20 mg/kg) was intraperitoneally injected prior to PM exposure for 1 h. (a) Representative images of lung sections stained with H&E. (b) The inflammation score for images of lung sections stained with H&E. (c) The number of the total cells, neutrophils, and macrophages in BALF. Values are the mean ± SEM; ^∗∗^*P* < 0.01, compared with the control group; ^#^*P* < 0.05 or ^##^*P* < 0.01, compared with the PM group; *n* = 5. EDA: edaravone; PM: particulate matter; BALF: bronchoalveolar lavage fluid.

To clarify the anti-inflammation and antioxidative effect of EDA on PM-induced mice, mice were treated with EDA (2, 10, or 20 mg/kg) before PM exposure every time. Our results showed that EDA could dose-dependently attenuate the infiltration of inflammatory cells in lung tissues of mice exposed to PM (Figures 3(a) and 3(b)). Furthermore, the increasing number of the total cell, neutrophils, and macrophages in BALF in the PM group was significantly inhibited by EDA pretreatment in a dose-dependent manner (Figure 3(c)). Besides, we found that alone EDA treatment in different concentrations has no toxic effect on the lung tissues and on blood cell numbers (Figure S1).

**Figure 4:**
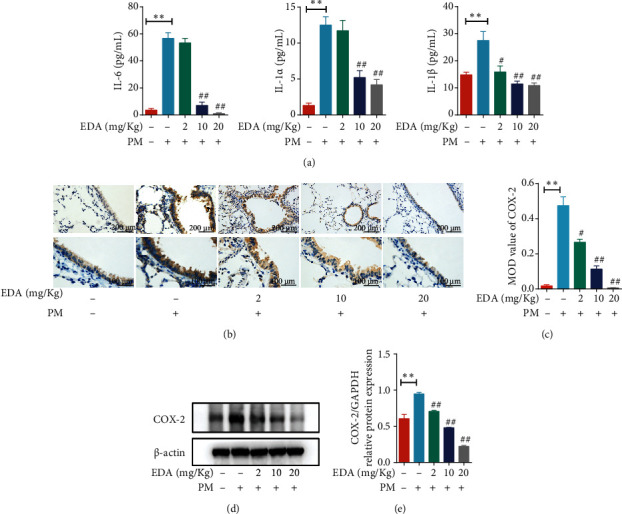
EDA attenuated the PM-induced inflammatory cytokines and protein expression in lung tissues. Mice were intratracheally instilled with PM at 100 *μ*g/day/mouse for 2 consecutive days. EDA (2, 10, or 20 mg/kg) was intraperitoneally injected prior to PM exposure for 1 h. (a) The levels of IL-6, IL-1*α*, and IL-1*β* in the BALF were measured by ELISA. (b) The level of COX-2 expression in lung tissues was measured by IHC staining. (c) The semiquantitative analysis was applied to compare the relative COX-2 protein expression. (d) COX-2 expression was determined by western blot. The optical densities of COX-2 are shown in (e). Values are the mean ± SEM;  ^∗∗^*P* < 0.01, compared with the control group; ^#^*P* < 0.05 or ^##^*P* < 0.01, compared with the PM group; *n* = 5. EDA: edaravone; PM: particulate matter; BALF: bronchoalveolar lavage fluid.

ELISA showed that EDA could attenuate PM-induced production of inflammatory cytokines IL-6, IL-1*α*, and IL-1*β* (Figure 4(a)). Meanwhile, the expression of COX-2 was analyzed using IHC, and we found that EDA significantly decreased PM-induced expression of COX-2 in bronchial epithelium in a dose-dependent manner (Figures 4(b) and 4(c)). Similarly, we tested the expression of COX-2 in lung tissues using western blot and showed that EDA treatment inhibited the expression of COX-2 in lung tissues of mice exposed to PM (Figures 4(d) and 4(e)). Besides, we found alone EDA treatment had no effect on inflammatory and COX-2 expression in normal lung tissues (Figure S2). These results indicated that EDA could significantly inhibit PM-induced lung inflammation *in vivo*.

### 3.4. EDA Decreased PM-Induced Oxidative Stress *In Vivo*

**Figure 5:**
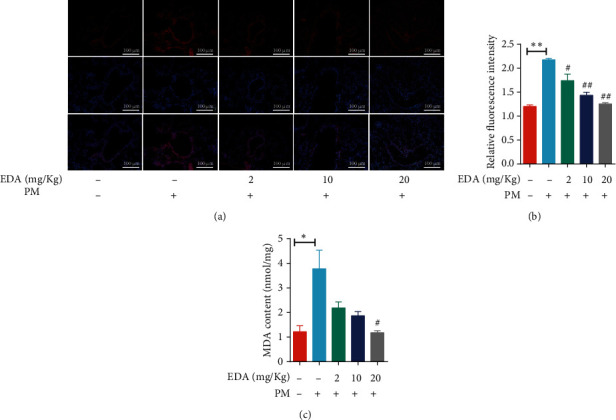
EDA inhibited the PM-induced oxidative stress in lung tissues. Mice were intratracheally instilled with PM at 100 *μ*g/day/mouse for 2 consecutive days. EDA (2, 10, or 20 mg/kg) was intraperitoneally injected prior to PM exposure for 1 h. (a) ROS levels in lung tissues were examined by DHE staining. Relative DHE fluorescence is shown in (b). (c) MDA levels were measured in the lung tissues. Values are the mean ± SEM; ^∗^*P* < 0.05 or ^∗∗^*P* < 0.01, compared with the control group; ^#^*P* < 0.05 or ^##^*P* < 0.01, compared with the PM group; *n* = 3 or 5. EDA: edaravone; PM: particulate matter.

The ROS level in lung tissues was detected with DHE staining and MDA activity. The ROS level in lung tissues of the PM-induced mouse model was significantly increased, and EDA treatment significantly reduced the PM-induced ROS generation in a dose-dependent manner (Figures 5(a) and 5(b)). Furthermore, the levels of MDA in lung tissues were also significantly increased in the PM group compared with those in the control group. EDA dose-dependent treatment significantly attenuated the levels of MDA induced by PM exposure (Figure 5(c)). These results showed that the levels of ROS in lung tissues were inhibited by EDA treatment in PM-exposed mice.

### 3.5. EDA Inhibited the Activation of the NF-*κ*B p65 Pathway in PM-Induced Inflammatory Responses

**Figure 6:**
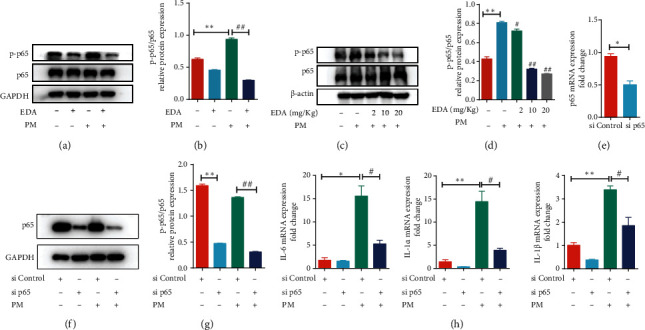
EDA attenuated PM-induced activation of NF-*κ*B p65 signaling pathway. (a) HBECs were treated with 500 *μ*M EDA and then stimulated with 300 *μ*g/mL PM for 24 h. The phosphorylation of the NF-*κ*B p65 signaling pathway was detected by western blot. The optical densities of phospho-NF-*κ*B p65 are shown in (b). (c) Mice were intratracheally instilled with PM at 100 *μ*g/day/mouse for 2 consecutive days. EDA (2, 10, or 20 mg/kg) was intraperitoneally injected prior to PM exposure for 1 h. The phosphorylation of NF-*κ*B p65 in lung tissues was detected by western blot. The optical densities of phospho-NF-*κ*B p65 are shown in (d). (e) The transfection efficiency of NF-*κ*B siRNA was detected by RT-PCR. (f) The protein level of NF-*κ*B was tested by western blot in HBECs which were transfected with NF-*κ*B siRNA with or without PM exposure. The optical densities of phospho-NF-*κ*B p65 are shown in (g). (h) The mRNA level of inflammatory factors IL-6, IL-1*α*, and IL-1*β* was detected by RT-PCR. ^∗^*P* < 0.05 or ^∗∗^*P* < 0.01, compared with the control group; ^#^*P* < 0.05 or ^##^*P* < 0.01, compared with the PM group; *n* = 3 or 5. EDA: edaravone; PM: particulate matter; HBECs: human bronchial epithelial cells.

To investigate the effect of EDA on the NF-*κ*B p65 pathway in PM-induced inflammatory responses, western blot was used the test the activation of the NF-*κ*B p65 pathway. We found that pretreatment with EDA blocked PM-induced activation of the NF-*κ*B p65 pathway in HBECs (Figures 6(a) and 6(b)). We also found that EDA treatment significantly decreased the expression of phospho-NF-*κ*B p65 in PM-induced lung inflammation in a dose-dependent manner (Figures 6(c) and 6(d)). Besides, we performed a knockdown of NF-*κ*B p65 by transfection with NF-*κ*B p65 siRNA in HBECs to further clarify the key role of the NF-*κ*B p65 signaling pathway in PM-induced inflammation. The efficiency of NF-*κ*B p65 siRNA was examined by RT-PCR and western blot. We found a decrease in the expression of NF-*κ*B p65 in HBECs after transfection with NF-*κ*B p65 siRNA with or without PM exposure (Figures 6(e)–6(g)). Furthermore, we found that knockdown of NF-*κ*B p65 significantly attenuated the production of inflammatory cytokines, including IL-6, IL-1*α*, and IL-1*β* in PM-exposed HBECs (Figure 6(h)). These results suggested that NF-*κ*B p65 signaling pathway activation played a vital role in PM-induced inflammation and EDA treatment could inhibit the activation of the NF-*κ*B p65 signaling pathway to alleviate PM-induced inflammation.

## 4. Discussion

**Figure 7:**
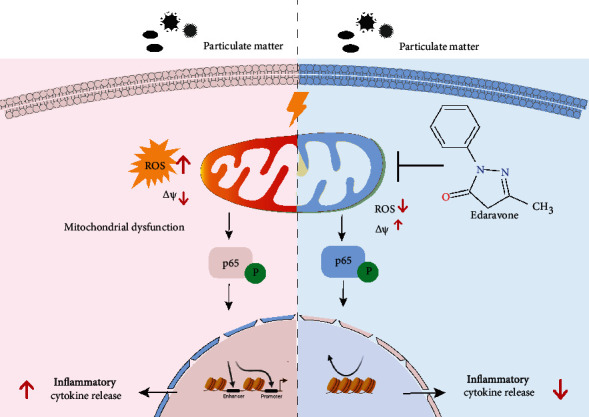
Schematic of the protective effect of EDA on PM-induced inflammation.

Inflammation is the primary biological mechanism of PM-induced lung damage and is responsible for the PM-induced development and exacerbation of chronic respiratory diseases. In this study, we showed that PM exposure triggered mitochondrial damage with the rapid increase in ROS and loss of MMP and thus promoted inflammatory cytokines and COX-2 expression in HBECs. EDA as the antioxidative drug decreased ROS production, reversed the MMP, and further inhibited the activation of the NF-*κ*B p65 signaling pathway in PM-induced HBECs and lung tissues (Figure 7). To our knowledge, this is the first study to demonstrate the protective effect of EDA on PM-induced lung inflammation.

EDA is clinically approved for treating stroke and amyotrophic lateral sclerosis (ALS) in previous researches by removing hydroxyl radicals. It can also modulate inflammatory processes, matrix metalloproteinase levels, nitric oxide production, apoptotic cell death, and necrotic cell death [11, 16, 17]. Tajima et al. found that EDA prevented lung injury and attenuated inflammatory cell activation and the release of inflammatory cytokines (IL-6 and TNF-*α*) in the BALF of the LPS-induced ALI mouse model [29]. It was also reported that EDA attenuated LPS-mediated inflammatory response in astrocytes [30]. Thus, EDA might be a powerful drug for inhibiting inflammatory responses. However, the protective effects of EDA on PM-induced inflammatory responses in the lung were unknown so far. In this study, we used PM-induced HBECs and mice to investigate the anti-inflammatory role of EDA. Our results provided direct evidence that EDA protected against PM-induced inflammation in HBECs and lung tissues of mice in a dose-dependent manner.

The signs of mitochondrial metabolic dysfunction included the increased ROS and loss of the membrane potential difference between the inner and outer membranes, which were also the hallmark of apoptotic mitochondrial damage [31]. Mitochondria constitute a significant source of ROS, affecting inflammatory response processes [32]. Previous studies showed that oxidative stress participated in regulating PM-induced inflammatory response, cell apoptosis, and even lung injury [33, 34]. Ning et al. found that the expression of antioxidant enzymes, superoxide dismutase 2 (SOD2), SOD3, and peroxiredoxin 4 (PRDX4) was decreased in PM-exposed lung tissues of mice [35]. Similar to these previous studies, we detected that PM exposure decreased MMP and further promoted ROS production in HBECs. EDA exhibits beneficial effects on free radical scavenging and maintaining the stability of MMP. Li et al. found that EDA treatment could increase ATP and mitochondrial membrane potential levels by augmenting the expression of Nrf2 and the target genes in excessive evaporation-induced dry eyes [36]. Reyes et al. found that EDA inhibited ischemia-reperfusion injury-induced pulmonary dysfunction by suppressing MDA levels and the expression of inflammatory cytokines [37]. Similarly, we firstly found that EDA could inhibit PM-induced mitochondrial imbalance and ROS production *in vitro* and *in vivo*. Elimination of oxidative stress may be a strategy to prevent PM-induced inflammation.

The NF-*κ*B pathway plays a crucial role in inflammatory responses by regulating the transcription of inflammatory cytokine genes [38]. Hassanein et al. demonstrated that EDA prevented cyclophosphamide-induced hemorrhagic cystitis and increased MDA by suppressing the NF-*κ*B signaling pathway in rats [39]. We also reported that PM exposure induced the activation of the NF-*κ*B pathway and increased the production of IL-6, IL-8, IL-1*β*, and COX-2 in HBECs [18]. In this study, we further demonstrated that the NF-*κ*B pathway was also significantly activated in PM-induced lung tissues of mice. Knockdown of NF-*κ*B p65 significantly inhibited the expression of inflammatory cytokines induced by PM exposure in HBECs.

Besides, the dose of PM exposure and EDA treatment in our studies was identified according to the previously reported studies [11, 17, 18, 23–27]. Based on the human exposure dose equivalent to the mouse-aspirated dose covert algorithm [40], we found that 100 *μ*g PM_2.5_ exposure for mice via intratracheal instillation was equivalent to 141 *μ*g/m^3^ PM_2.5_ exposure for humans in 24 hours. According to the World Health Organization (WHO) air quality guidelines, the recommended short-term (24-hour) AQG level of interim target-1 of PM_2.5_ is 75 *μ*g/m^3^. Thus, the dose of PM exposure for mice in our study could contribute to lung damage. To make our results more accurate, we selected the concentration gradient of EDA for our study. EDA has been widely used in clinical treatment of acute cerebral infarction, and the recommended dose of EDA was 30 mg twice a day according to the drug instruction. According to the drug dose convert algorithm between humans and mice based on the body surface area [41], the concentration of EDA in our study was equivalent to 0.163, 0.813, and 1.626 mg/kg for humans. Thus, the concentration of EDA for the clinical treatment was included in our concentration range in this study. Furthermore, we demonstrated that alone EDA treatment with different concentrations had no toxic effect in our study.

## 5. Conclusion

Taken together, we firstly found that EDA inhibited the expression of IL-6, IL-1*α*, IL-1*β*, and COX-2 production in HBECs and mice exposed to PM. Meanwhile, EDA attenuated PM-induced mitochondrial dysfunction, ROS overproduction, and activation of the NF-*κ*B signaling pathway. We demonstrated that EDA could significantly alleviate PM-induced lung inflammation by inhibiting the ROS-NF-*κ*B p65 signaling pathway *in vitro* and *in vivo*. These data provided evidence that EDA would be a beneficial therapeutic choice in preventing and treating the harmful effects of PM exposure in the future.

## Figures and Tables

**Table 1:** Primers used in the study.

Genes	Forward	Reverse
GAPDH	5′-AAGGTGAAGGTCGGAGTCAAC-3′	5′-GGGGTCATTGATGGCAACAATA-3′
COX-2	5′-TAAGTGCGATTGTACCCGGAC-3′	5′-TTTGTAGCCATAGTCAGCATTGT-3′
IL-6	5′-ACTCACCTCTTCAGAACGAATTG-3′	5′-CCATCTTTGGAAGGTTCAGGTTG-3′
IL-1*α*	5′-TGGTAGTAGCAACCAACGGGA-3′	5′-ACTTTGATTGAGGGCGTCATTC-3′
IL-1*β*	5′-ATGATGGCTTATTACAGTGGCAA-3′	5′-GTCGGAGATTCGTAGCTGGA-3′

## Data Availability

All supporting the findings of this report are included in this article. Source raw data for all quantitations is fully available upon request.

## Supplementary Materials

Supplementary Materials Figure S1: alone EDA treatment had no toxic effect on lung tissues. Figure S2: alone EDA treatment had no effect on the expression of inflammatory cytokines and protein expression.
