# Supplementary material for: Edaravone Attenuated Particulate Matter-Induced Lung Inflammation by Inhibiting ROS-NF-κB Signaling Pathway
Source: Oxid Med Cell Longev. 2022 Apr 23;2022:6908884. doi: 10.1155/2022/6908884 (PMC9056219; doi:10.1155/2022/6908884)
Supplement: Supplementary Materials — Figure S1: alone EDA treatment had no toxic effect on lung tissues. Figure S2: alone EDA treatment had no effect on the expression of inflammatory cytokines and protein expression. [file 6908884.f1.zip › Supplementary Materials 20220327.docx]

**Edaravone attenuated particulate matter-induced lung inflammation by inhibiting ROS-NF-κB signaling pathway**


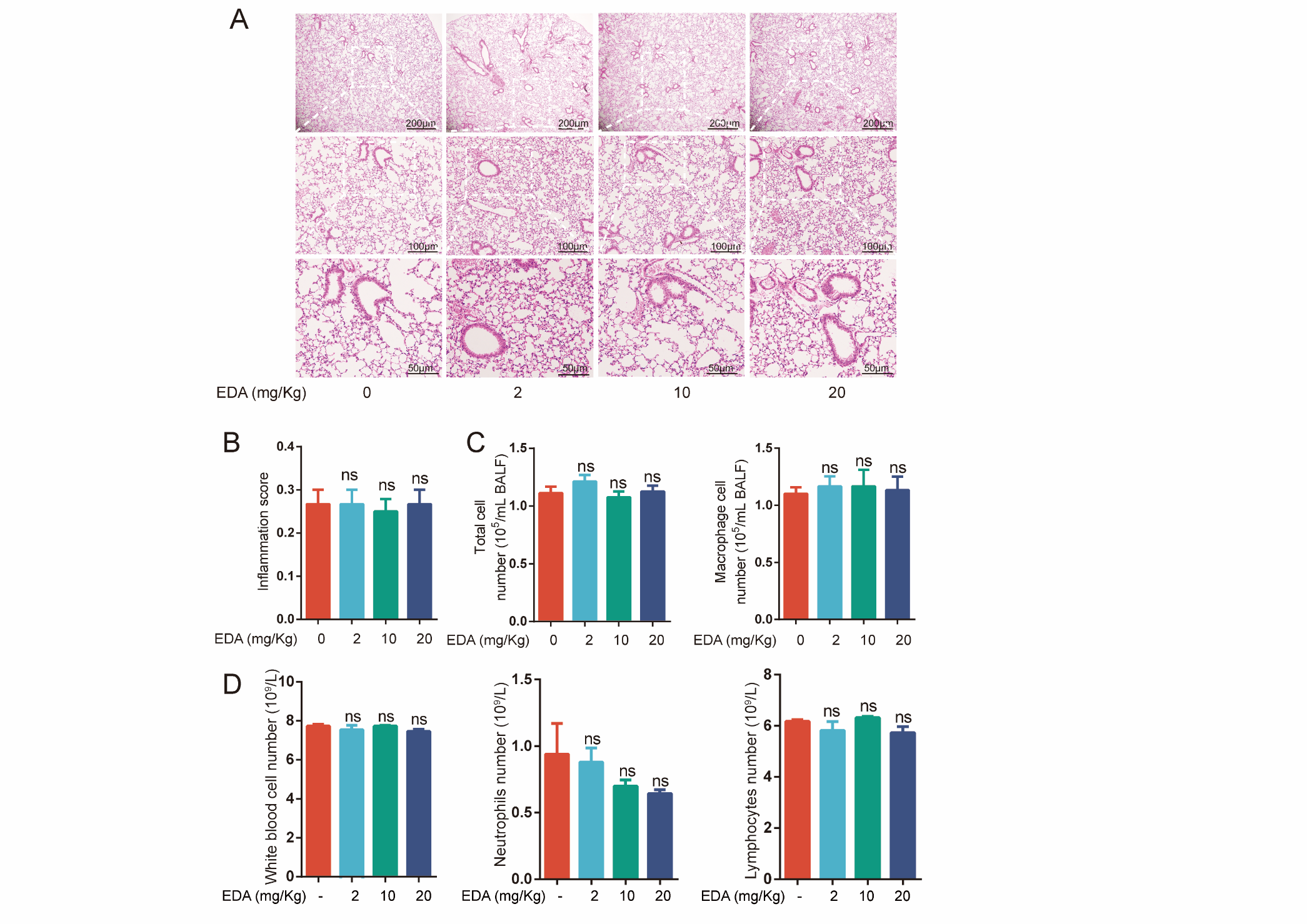


**Figure S1. Alone EDA treatment had no toxic effect on lung tissues.** EDA (2, 10, or 20 mg/Kg) was intraperitoneally injected for 2 consecutive days. (A) Representative images of lung sections stained with H&E. (B) The inflammation score for images of lung sections stained with H&E. (C) The number of the total cells and macrophages in BALF. (D) The number of the white blood cells, neutrophils and lymphocytes in whole blood. Values are the mean ± SEM; ns, no significance, compared with the control group; n=3. EDA, edaravone; BALF, bronchoalveolar lavage fluid.


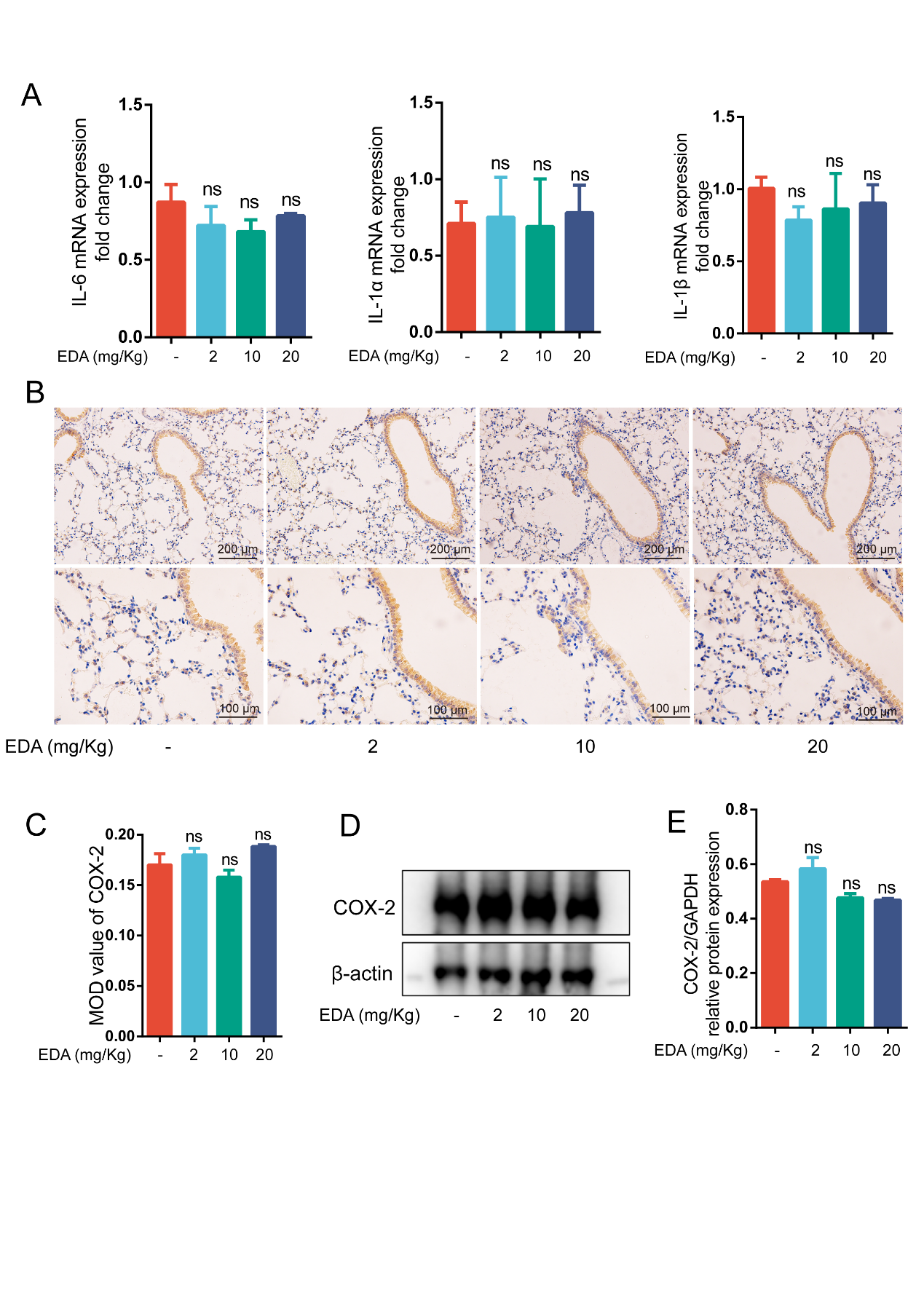


**Figure S2. Alone EDA treatment had no effect on the expression of inflammatory cytokines and proteins expression.** EDA (2, 10, or 20 mg/Kg) was intraperitoneally injected for 2 consecutive days. (A) The levels of IL-6, IL-1α, and IL-1β in lung tissues were measured by RT-PCR. (B) The level of COX-2 expression in lung tissues was measured by IHC staining. (C) The semi-quantitative analysis was applied to compare the relative COX-2 protein expression. (D) COX-2 expression was determined by western blot. The optical densities of COX-2 are shown in (E). Values are the mean ± SEM; ns, no significance, compared with the control group; n=3. EDA, edaravone.
