# Supplementary figures and images for: Edaravone Attenuated Particulate Matter-Induced Lung Inflammation by Inhibiting ROS-NF-κB Signaling Pathway
Source: Oxid Med Cell Longev. 2022 Apr 23;2022:6908884. doi: 10.1155/2022/6908884 (PMC9056219; doi:10.1155/2022/6908884)

A

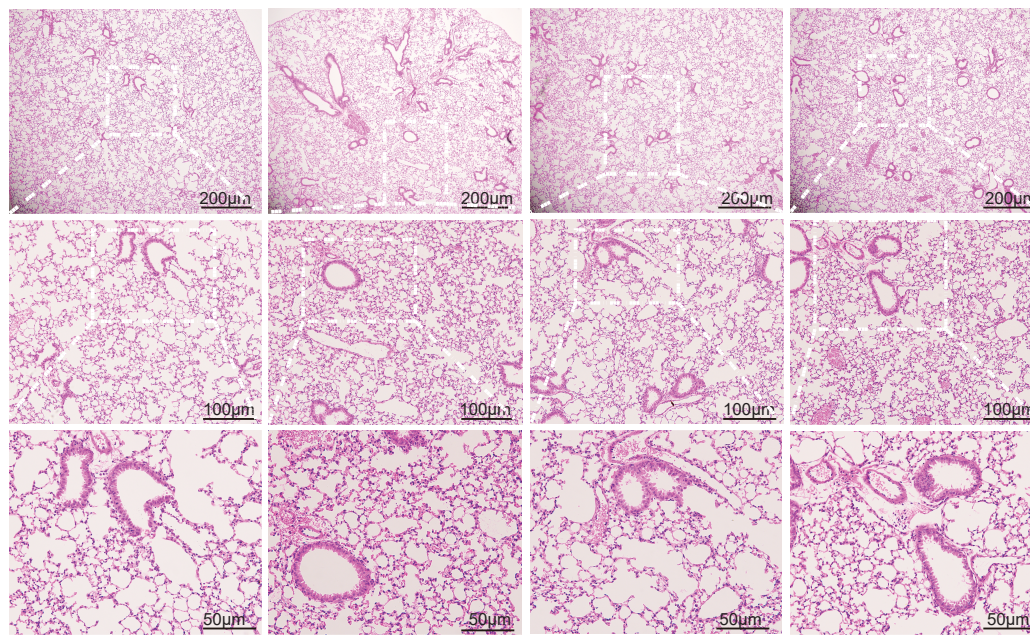

EDA (mg/Kg)      0                      2                      10                      20

B

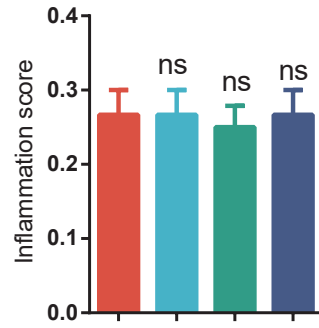

EDA (mg/Kg)

C

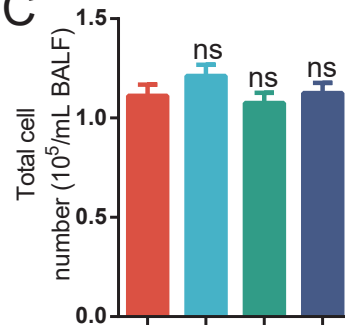

EDA (mg/Kg)

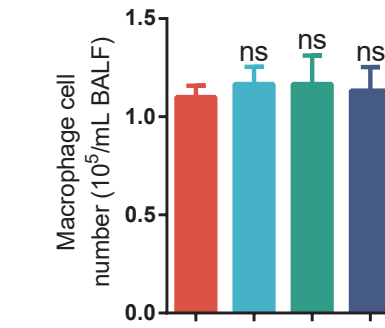

EDA (mg/Kg)

D

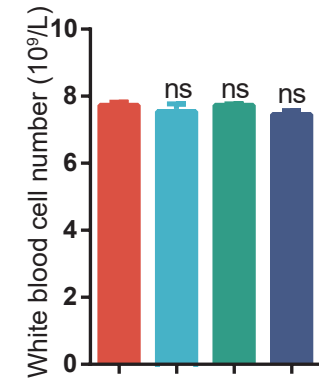

EDA (mg/Kg)

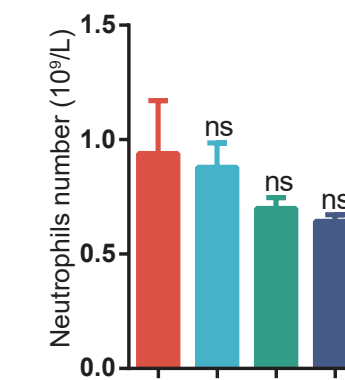

EDA (mg/Kg)

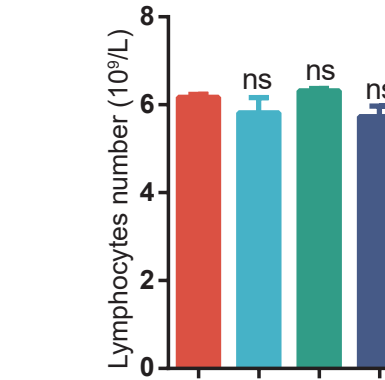

EDA (mg/Kg)

Supplement: Supplementary Materials — Figure S1: alone EDA treatment had no toxic effect on lung tissues. Figure S2: alone EDA treatment had no effect on the expression of inflammatory cytokines and protein expression. [file 6908884.f1.zip › Supplementary Figure 1.pdf]

**A**

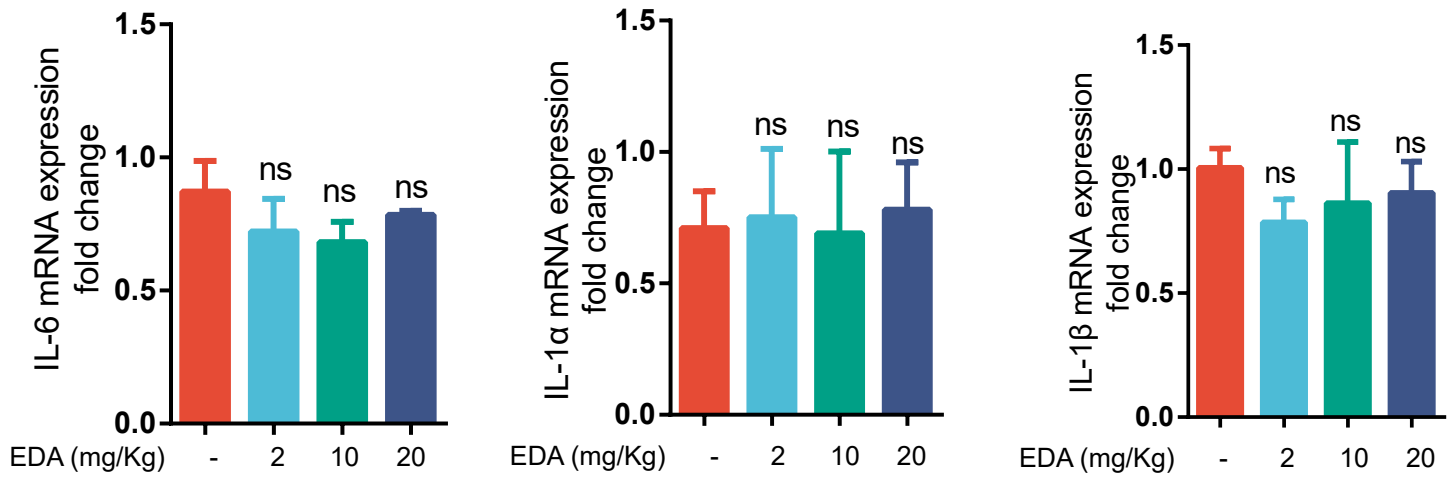

B

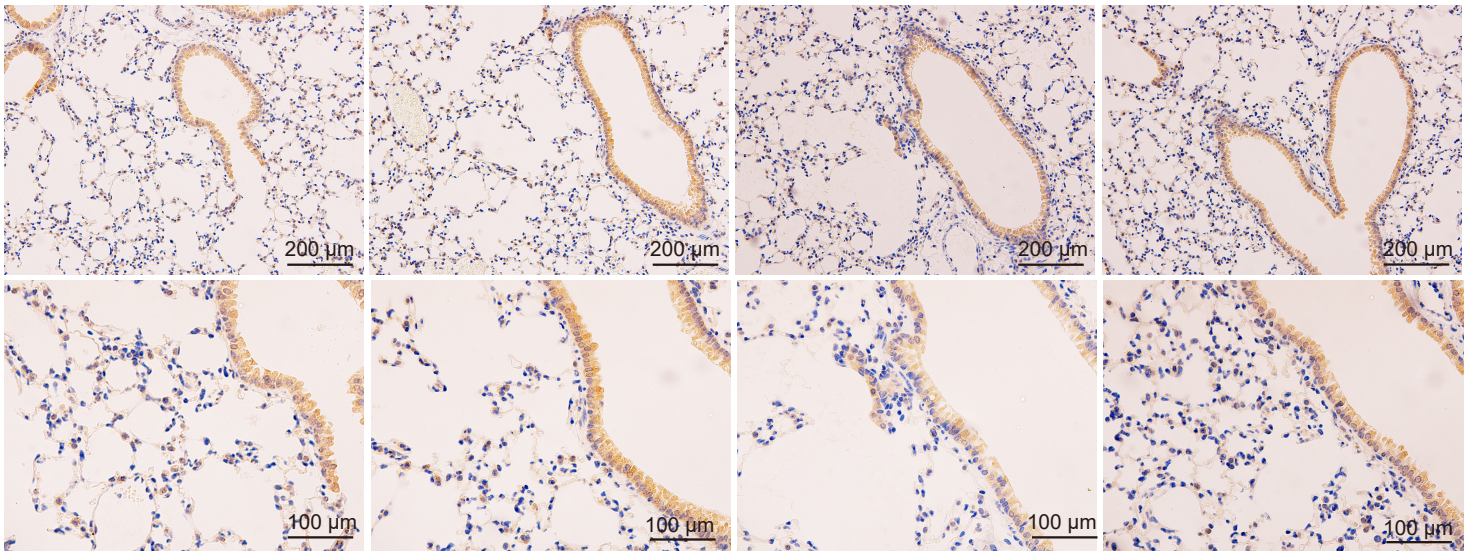

|             |   |   |    |    |
|-------------|---|---|----|----|
| EDA (mg/Kg) | - | 2 | 10 | 20 |
|-------------|---|---|----|----|

C

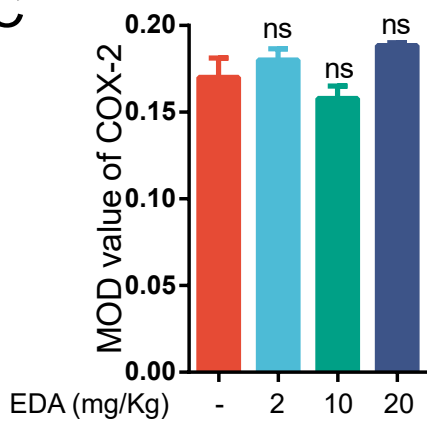

D

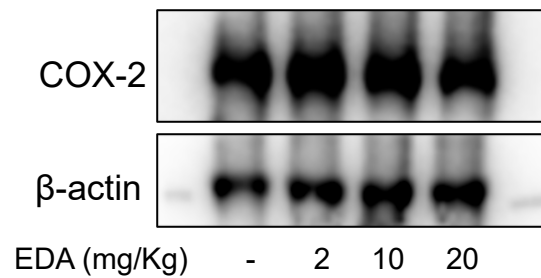

# E

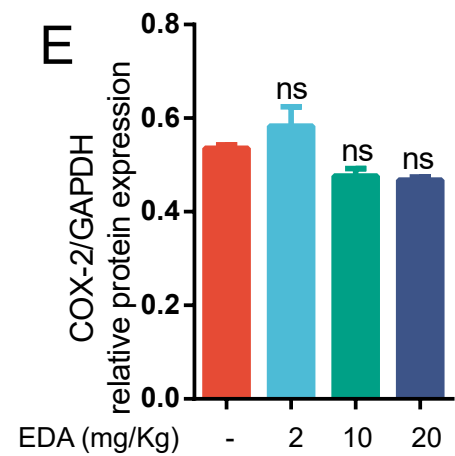

Supplement: Supplementary Materials — Figure S1: alone EDA treatment had no toxic effect on lung tissues. Figure S2: alone EDA treatment had no effect on the expression of inflammatory cytokines and protein expression. [file 6908884.f1.zip › Supplementary Figure 2.pdf]
